# On the attractiveness of working as a GP and rural doctor including admission pathways to medical school – results of a German nationwide online survey among medical students in their “Practical Year”

**DOI:** 10.3205/zma001498

**Published:** 2021-09-15

**Authors:** Susan Selch, Stefanie Pfisterer-Heise, Wolfgang Hampe, Hendrik van den Bussche

**Affiliations:** 1 University Medical Centre Hamburg-Eppendorf, Institute of Biochemistry and Molecular Cell Biology, Hamburg, Germany; 2 University Medical Centre Hamburg-Eppendorf, Institute and Polyclinic of General Medicine, Hamburg, Germany

**Keywords:** student selection medicine, general practice, primary care, GP, rural doctor quota

## Abstract

**Background: **One of the aims of the German student selection network (Studierendenauswahl-Verbund, stav) is to review existing procedures for selecting medical students and to relate their effectiveness to students’ career aspirations as well as to their further careers. Against the background of changes in the selection procedures and the introduction of the rural doctor quota (Landarztquote), the study conducted here aims at contributing to the current discussion on the future of GP (general practitioners) care, especially in rural areas.

**Methods:** In 2019 and 2020, the stav conducted a German nationwide online survey among medical students towards the end of their “Practical Year” (Praktisches Jahr, final-year medical students in practical training). The associations between selection parameters and students’ interest in later working as a GP as well as students’ preference to later work in a place with a low population density were investigated. Furthermore, socio-demographic variables and variables related to medical studies were taken into account. Statistical comparisons were carried out using Chi^2^- and Mann-Whitney U tests.

**Results: **A total of 1,055 students in their Practical Year (65.4% female, 27 years) completed the survey. As their final professional position, 12.1% aspired to own a GP practice or work as employed GP after completing medical specialist training in general medicine (interested students: 9.9%) or general internal medicine (interested students: 9.5%). Compared to their fellow students, those interested in working as a GP had been more often admitted to medical school via the waiting time quota and had more often already completed vocational training in a medical-related field. 39.1% of those interested in working as a GP wanted to work in a place with a low population density. Coming from a place with a low population density as well as completing the medical internship (Famulatur) for GP care in such a place turned out to be positive influencing factors.

**Discussion: **The observed associations between waiting time quota and interest in working as a GP as well as between origin from a place with a low population density and preferring to later work in such a place go hand in hand with changes in the access regulations for medical studies, which concern both the waiting time quota (abolition of the latter) and a regulation of the number of rural doctors (rural doctor quota). In order to evaluate the current changes in the access regulations for medical studies, longitudinal studies are desirable that cover the time from the application to study up to the medical specialist examination and further career.

## Zusammenfassung

**Hintergrund: **Der Studierendenauswahl-Verbund (stav) hat u.a. das Ziel, bestehende Instrumente zur Auswahl Medizinstudierender zu überprüfen und die Aussagekraft der Auswahlverfahren in Beziehung zu setzen zur angestrebten Berufstätigkeit bzw. zum Berufsverbleib. Vor dem Hintergrund der Neuerungen der Auswahlverfahren und Einführung der Landarztquote soll die hier durchgeführte Studie einen Beitrag leisten zur aktuellen Diskussion um die zukünftige hausärztliche Versorgung, v.a. in ländlich geprägten Gebieten.

**Methoden:** In 2019 und 2020 führte der stav eine bundesweite Onlineumfrage unter Medizinstudierenden gegen Ende ihres PJ durch. Es wurde untersucht, welcher Zusammenhang zwischen Auswahlparametern und dem Wunsch besteht, eine hausärztliche Tätigkeit anzustreben und in einem Ort mit geringer Bevölkerungsdichte zu arbeiten. Weiterhin wurden soziodemographische Faktoren und Merkmale zum Studium berücksichtigt. Statistische Vergleiche erfolgten mittels Chi-Quadrat- und Mann-Whitney-U-Tests.

**Ergebnisse: **Insgesamt 1.055 PJ-Studierende (65,4% weiblich, 27 Jahre) schlossen die Umfrage ab. Als endgültige berufliche Position erstrebten 12,1% eine Niederlassung oder eine Anstellung als Hausärzt*in nach absolvierter Weiterbildung in Allgemeinmedizin (interessierte PJ-Studierende: 9,9%) oder Innere Medizin ohne Schwerpunkt (interessierte PJ-Studierende: 9,5%). Im Vergleich zu ihren Kommiliton*innen hatten die Hausarztinteressierten ihren Studienplatz überzufällig häufig über die Wartezeitquote erhalten und hatten häufiger bereits eine medizinnahe Berufsausbildung abgeschlossen. In einem Ort mit geringer Bevölkerungsdichte zu arbeiten wünschten sich 39,1% der Hausarztinteressierten. Die Herkunft aus einem Ort mit geringer Bevölkerungsdichte sowie eine Ableistung der Famulatur zur hausärztlichen Patientenversorgung in einem solchen Ort stellten sich hier als positive Einflussfaktoren heraus.

**Diskussion:** Die beobachteten Zusammenhänge zwischen Wartezeitquote und Hausarztpräferenz sowie zwischen Herkunft aus einer dünn besiedelten Region und Landarztpräferenz gehen zeitlich einher mit Veränderungen in den Zugangsregelungen zum Medizinstudium, die sowohl die Wartezeitquote (Abschaffung dieser) als auch eine Regulierung der Landarztzahlen (Landarztquote) betreffen. Um die aktuellen Veränderungen der Zugangsregelungen zum Medizinstudium zu evaluieren, sind längsschnittliche Studien erstrebenswert, die die Zeit von der Studienbewerbung bis hin zur fachärztlichen Prüfung und dem Ort der Tätigkeit umfassen.

## 1. Background

Only a few studies are available for the German-speaking countries that examine the associations between the admission pathways to medical school and students’ desired and actual careers. Rarely have selection parameters such as the Abitur grade (German general higher education entrance qualification), admission rates or professional experience been related to later professional activity. Career choices of young doctors are of interest because of the imbalance in the sectoral and regional distribution of doctors in Germany. In particular, there has been a lack of doctors in GP care in rural areas and in the public health system for years [1], and surgery is also increasingly complaining about falling numbers of young doctors [2]. In addition, GP posts are increasingly going unfilled even in large cities, so that the shortage of GPs is no longer limited to rural areas [3].

Medical students in Germany decide primarily according to their personal interests which specialist training they will choose after completing their undergraduate studies and where they want to work later. The societal need for healthcare, demographic changes or other external factors play a minor role [4], [5]. In order to ensure GP care in the future, new models are being developed that include telemedicine and physician delegation of clinical tasks. Further measures will start during undergraduate education. For example, one of the goals of the Master Plan for Medical Studies 2020 (Masterplan Medizinstudium 2020) of the German Federal Ministry of Education and Research is to strengthen general medicine in education and training [6] which also opens up the possibility of a “rural doctor quota“ ([6], p.12). This allows federal states to reserve study places for students who will complete specialist training in general medicine and then work for 10 years in an underserved region. The state of North Rhine-Westphalia was the first to introduce such a quota for the 2019/20 winter semester [7]. Bavaria [8], Saxony-Anhalt [9], Rhineland-Palatinate [10] and Saarland [11] followed as of the winter semester 2020/21. Since then, between 5% and 7.8% of medical study places have been reserved for the rural doctor quota in these five federal states. Baden-Württemberg decided to create 150 new medical study places for the winter semester 2020/21, 75 of which will go to future rural doctors [12]. Other federal states, such as Mecklenburg-Western Pomerania and Lower Saxony, are planning to introduce the rural doctor quota from the winter semester 2021/22.

The rural doctor quota comes into force at a time when the procedure for selecting medical students is changing fundamentally. On 19 December 2017, the German Federal Constitutional Court (Bundesverfassungsgericht, BVerfG) ruled that essential contents of the procedure for allocating places in medical studies were in part not compatible with the German Basic Law [13]. In a subsequent jointly agreed state treaty, the federal states established a reform of the selection criteria from the summer semester 2020 [14]. This includes, among other things, an increase in the Abitur top quota (Abiturbestenquote) from 20% to 30%. The waiting time quota, by means of which 20% of places in medical studies were previously allocated, will be abolished. 60% of the study places will continue to be awarded via the selection procedures of the universities (Auswahlverfahren der Hochschulen, AdH). A subject-specific aptitude test is a compulsory part of this. New is the introduction of the additional aptitude quota (Zusätzliche Eignungsquote, ZEQ, 10%), for which aptitude tests can also be used.

One of the few studies in the German-speaking countries that has so far examined the association between selection parameters and career aspirations is the study by Kesternich et al. [15]. The authors surveyed more than 1,300 Munich medical students in 2012 and 2014 and found that the desire to work as a rural doctor correlates significantly positively with relatively low Abitur grades. Furthermore, a higher (self-reported) risk aversion and the fact that at least one parent is a doctor increased the probability of preferring to work as a GP. The factor of rural origin, which is found in many international studies to be a prognostic factor for a later occupation as a rural doctor [16], [17], was not examined by Kesternich et al. [18].

## 2. Study aim

Against the background of the changes in the selection procedures and the introduction of the rural doctor quota, the study conducted here is intended to contribute to the current discussion on GP care, especially in rural areas. It examines the association between selection parameters and Practical Year students’ interest in working as a GP as well as their preference to work in a small town (<20,000 inhabitants) or rural region. Small towns and villages are often grouped together as forms of settlement in rural areas and are also referred to in the following as a “place with a low population density”. In addition to the influence of selection parameters, other factors such as gender, doctor as a parent are taken into account. Unlike in the study by Kesternich et al., regional origin is also included. Students in their Practical Year from all over Germany were invited to participate in the survey. The study was designed within the German student selection network (stav; [https://www.projekt-stav.de/]). One of the aims of the stav is to review existing instruments for selecting medical students and to relate their effectiveness to students’ career aspirations as well as their further careers.

## 3. Methods

### 3.1. Sample

In October 2019 and March 2020, all medical faculties in Germany were contacted by the German Association of Medical Faculties (Medizinischer Fakultätentag) with the request to recruit their medical students towards the end of their Practical Year to participate in the study and to forward the corresponding survey link to them. 

1,111 students participated in the survey (N=772 from 10/19–01/20; N=339 from 03–07/20). Before the start of the survey, students agreed to participate in the study and had the opportunity to provide an e-mail address at which they expected to be reachable after completing their studies. The aim of the stav is to conduct a new survey after about 1.5 years in order to examine, among other things, the implementation of students’ career aspirations stated in this survey. Ten ipads were raffled among all participants.

#### 3.2. Survey instruments

Questions on career aspirations were taken from the survey instruments of the longitudinal KarMed observation study (2008-2015) [19]. Here, students were asked which specialist training they would like to pursue after completing their studies, in which sector of care they would like to finally work (own a GP practice, own a specialist practice, be employed as a GP, be employed as a specialist, specialist in a hospital, senior physician in a hospital, chief physician in a hospital, I don’t know [yet], other) and in which size of town they would prefer to work in the future (in a large city [>100,000 inhabitants], in a medium-sized town [20,000 - 100,000 inhabitants], in a small town [<20,000 inhabitants], in a rural region, I don’t know [yet]). The following socio-demographic variables were collected: age, gender, origin, parenthood, doctor as parent and whether vocational training in a medical-related field was completed. In addition, the respondents were asked to provide information on Abitur grade, admission pathway to medical school, place of study, type of study program and performance in the state examinations. They were also asked about the size of the town in which they completed their medical internship for GP care (in a large city [>100,000 inhabitants], in a medium-sized town [20,000 - 100,000 inhabitants], in a small town [<20,000 inhabitants], in a rural region). Attitudes towards life and working as a GP in rural areas were recorded using the questionnaire by Steiner-Hofbauer et al. [20], in which the response format consists of a five-point Likert scale from 1 (Strongly disagree) to 5 (Strongly agree). The survey was conducted online. The survey software used was limesurvey, version 2.62.2.

#### 3.3. Statistical analyses

The questions were mostly compulsory. Not answering facultative questions was counted as missing values. For the statistical analyses, only data sets of participants who had completed the survey were considererd. Statistical analyses were conducted using SPSS for Windows version 26. For the bivariate analysis, contingency tables were created and the stochastic independence was checked by Chi-square tests. The phi coefficient (*φ*) is given here as a measure of the effect size. Furthermore, Mann-Whitney U tests were carried out for independent samples, for which the correlation coefficient (*r*) indicates the measure of effect size. Abitur grades and Physikum/M1 (first part of medical physician’s examination) grades included in the analyses were standardised by subtracting the mean and dividing by the standard deviation.

#### 3.4. Ethics approval

The Local Psychological Ethics Committee (LPEK) at the Centre for Psychosocial Medicine of the University Medical Centre Hamburg-Eppendorf (UKE) has approved the study (LPEK-0042). 

## 4. Results

### 4.1. Study cohort

**Figure 1:**
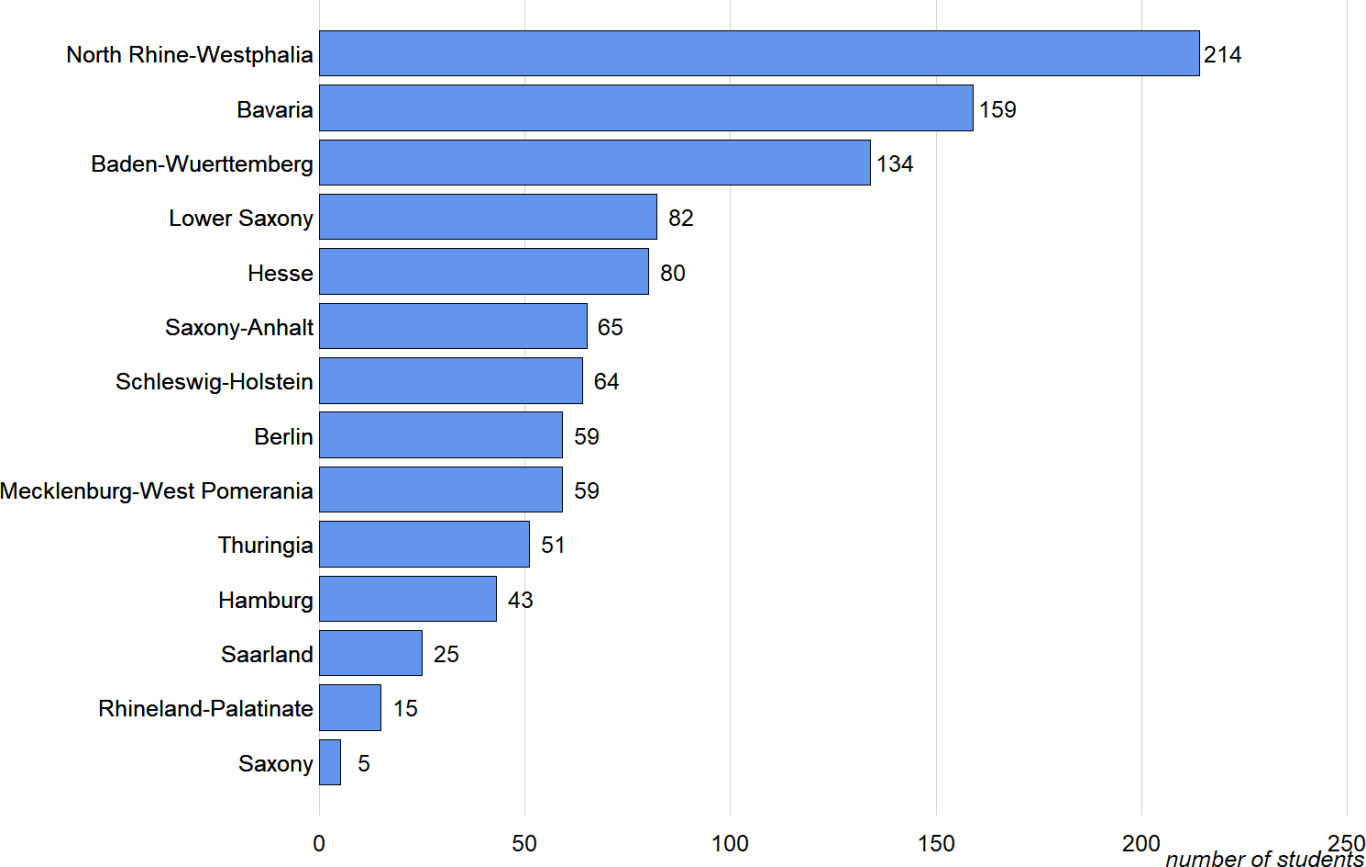
Study participants by state (total N=1,055).

Of the 1,111 students in their Practical Year, 1,055 completed the survey. They can be assigned to 35 medical faculties and 14 federal states (see figure 1 (Fig. 1)). 56 participants dropped out of the survey prematurely. Their answers were not included in the analyses. Among the responses of those who completed the survey, only a few missings were recorded and were neglected (age: N=1, gender: N=2, vocational training: N=4, parenthood: N=5 and Physikum/M1 grades: N=49).

Of the 1,055 students, 690 were female (65.4%), i.e. slightly more (3%) than the average in medical studies (2019 student figures [21]). The mean age was 27.2 years (SD=3.1, see table 1 (Tab. 1)). 614 (58.2%) of the respondents stated that they had received their place at university via the selection procedure of their university (AdH). 152 (14.4%) had obtained their place via the Abitur top quota, 129 (12.2%) via the waiting time quota, with an average waiting time of 13.4 semesters (SD=1.8). The remainder were distributed among others (N=97; 9.2%; e.g. study place via the German Armed Forces, lottery procedure), second degree (N=25; 2.4%), hardship case (N=2; 0.2%) or stated “I don’t know” (N=36; 3.4%).

#### 4.2. Interest in working as a GP

§ 73 par. 1 of the Fifth Social Code (SGB V) [22] regulates who is allowed to work as a GP in Germany. According to this, the following participate in GP care for adults: general practitioners and specialists in general internal medicine who have chosen to participate in GP care on the occasion of their outpatient work. Of the 1,055 respondents, 9.9% (N=104) were aiming for specialist training in general medicine, and a further 9.5% (N=100) for specialist training in general internal medicine. Of these 204, 128 (62.8%) wanted to work as a GP in the future (12.1% of the total group) – own a practice or be employed as a GP. 73% of those interested in working as a GP were female (see table 1 (Tab. 1)).

The comparison between those interested in working as a GP and those not interested shows that those with an interest were disproportionately likely to have been admitted to university via the waiting time quota (Χ^2^(1)=8.873; *p*<.01; *φ*=0.10). According to Cohen [23], this is a weak effect. Furthermore, these students more often than their fellow students who were not interested in working as a GP had already completed vocational training in a medical-related field, e.g. in health care and nursing or paramedicine (Χ^2^(1)=10.239; *p*=.001; *φ*=0.10). Here, too, a weak effect can be observed. Moreover, significant differences emerged with regard to age (U=51,167.5; *p*<.05; *r*=0.08) and location of medical internship for GP care (Χ^2^(1)=4.870; *p*<.05; *φ*=0.07), both with very low effect sizes. The group of students interested in working as GPs was further investigated in the following and considered in a differentiated manner with regard to location preference for the aspired career.

#### 4.3. Interest in working as a GP – differentiated by preference for location size

Of the 128 students interested in working as a GP in the future, 39.1% (N=50) said they wanted to work in a place with a low population density later on. 46.9% (N=60) preferred towns with more than 20,000 inhabitants and 14.0% (N=18) did not want to commit themselves yet. Characteristics of the students with an interest in working as a GP, differentiated according to location preference (except for the undecided), are shown in table 2 (Tab. 2).

In contrast to students with an interest in working as a GP and a preference for location size of more than 20,000 inhabitants, students with an interest in working as a GP who wanted to work in a region with a low population density were disproportionately likely to have grown up in such a region themselves (Χ^2^(1)=19.250; *p*<.001; *φ*=0.42). According to Cohen [23], this is a medium effect size. In addition, they were more likely to already have completed their medical internship for GP care in such a region (Χ^2^(1)=12.568; *p*<.001; *φ*=0.34), for which a medium effect could also be demonstrated. With a weak effect, it was shown that those interested in working as a GP with a location preference for more than 20,000 inhabitants were more frequently selected via the AdH procedure than those who wanted to work in a region with a low population density (Χ^2^(1)=4.365; *p*<.05; *φ*=0.20). Attitudes towards living and working as a GP were then contrasted for those interested in working as a GP with a preference for large vs. small towns (see table 3 (Tab. 3)).

Those interested in working as a GP and preferring to work in a place with a low population density, who consider living in a village community more desirable than their fellow students (U=753.5; *p*<.001; *r*=0.44; medium effect), agreed more that living in the country offers advantages for families (U=1,113.5; *p*<.05; *r*=0.23). This is a weak effect. Those interested in working as a GP and preferring to work in a place with a higher population density, for whom an urban infrastructure is more indispensable than for their fellow students (U=894.0; *p*<.001; *r*=0.36; medium effect), agreed more that a GP in the countryside has longer working hours than in the city (U=1,152.5; *p*<.05; *r*=0.21). This is a weak effect. With regard to all other aspects, there were no differences in the evaluation by the subgroups with different preferences for location size. 

## 5. Discussion

Of the medical students surveyed here who were about to complete their studies in Germany at the time of the study, 9.9% aspired to specialist training in general medicine, which corresponds to the results of comparable German surveys [24], [25], [26], [27]. The wish to work as a GP in the future was even expressed by 12.1% of all respondents, if the students opting for general internal medicine as their preferred specialist training were also taken into account.

### 5.1. Selection quotas

Compared to the students who were not interested in working as GPs later on, the students with an interest in working as a GP were more likely to have obtained their place at university via the waiting time quota and had more often already completed vocational training in a medical-related field. Both effects were weakly observed and cannot be interpreted independently of each other, because many applicants bridge the waiting period for a place at medical school by completing vocational training and subsequent employment. They are also correspondingly older. In contrast to Kesternich et al. [15], we found no influence of the Abitur grade or the fact that one parent is a doctor on being interested in working as a GP.

If those interested in working as a GP have disproportionately often been admitted to medical school via the waiting time quota, the question arises as to whether the abolition of the waiting time quota will lead to a decrease in the number of GPs in the future. It is possible that those who would like to study medicine and are interested in working as a GP in an underserved region will be admitted via the rural doctor quota. However, compared to those interested in working as a GP with a preference for a place with a higher population density, those interested in working as a GP in rural areas in our study did not state more frequently that they would have committed themselves to practicing in the countryside for a certain period of time if this had increased their chances of being admitted to medical school. It can be speculated that these students would have been more likely to seek their study place via the ZEQ or specific AdH sub-quotas. At most faculties, completed vocational training in a medical-related field (e.g. nursing) and/or a corresponding occupation are taken into account in AdH (sub)quotas and are included in the ZEQ in many faculties. The universities of Greifswald and Jena, for example, have set up AdH sub-quotas in which the criterion of recognized vocational training is even rated highest [28]. 

#### 5.2. Experience and regional origin

Among those interested in working as a GP, the preference for later working in a place with a low population density was more frequent among those who had already completed their medical internship for GP care in such a place. In addition to the fact that it is precisely the students with an interest in working as a rural doctor from the outset who completed their internship for GP care in a rural area, the early contact with GP practice in a rural region may also have increased their interest in wanting to work as a rural doctor later on. Possible prejudices against working as a rural doctor could perhaps be reduced, e.g. the assumption of longer working hours than in the city. Observational studies abroad have also shown that internships or training periods in rural practices, hospitals or social institutions lead to more doctors working in rural areas [29], [30]. In this respect, it is to be welcomed that in Germany, too, medical curricula are increasingly oriented towards primary care per se and towards GP care in rural regions [31], [32], [33]. 

Another significant factor for preferring to later work as a GP in a place with a low population density turned out to be students’ biographical origin from such a region. This is in accordance with the results of many foreign studies [16], [17], [34]. In Japan and Australia, for example, origin from rural regions is therefore also used as an admission criterion [35]. In Germany, this is not conceivable because of the Principle of Equality according to article 3 of the German basic law. 

## 6. Conclusion

The current changes in the admission regulations for medical studies are manifold. In addition to the abolition of the waiting time quota, faculty-specific ZEQ and AdH (sub)quotas as well as state-specific rural doctor quotas have created a wide variety of pathways for applicants to obtain a place at a medical school. In addition, faculties are given a great deal of leeway to determine and weight their own criteria for student selection. It remains to be seen how this will shape the composition of first-year students in the future and whether and how this will affect the career preferences of those with a certification to enter postgraduate medical education in Germany (Approbation). A strong intention to complete specialist training in general practice could be used as a criterion for validating the changes in the admission rules or to evaluate faculty-specific selection procedures. Longitudinal studies that cover the period from the application to study up to the medical specialist examination and careers afterwards are desirable [18]. 

### 6.1. Strengths and limitations

The strengths of the study are its multicentricity and the sample size. Due to the high number of participating students from different parts of Germany, this study is of great relevance for examining associations between selection processes and career aspirations. Limitations of the study are that it cannot be ruled out that non-participation in the study was based on systematic characteristics. This could have led to biased results. Furthermore, it should be noted that the data are self-assessments of the students surveyed at the time towards the end of their Practical Year and do not reflect students’ actual career choice. Also, changes in specialist preference are not unusual in the course of specialist training. For general medicine, it could be shown that more than half of the physicians are career changers towards the end of their specialist training in general medicine [36]. One aim of the stav is to follow the students surveyed here longitudinally in order to investigate, among other things, the implementation of career aspirations.

## Funding

The German student selection network (Studierendenauswahl-Verbund, stav) is funded by the Federal Ministry of Education and Research for the period 07.2018-12.2021 (Funding code 01GK1801A).

## Competing interests

The authors declare that they have no competing interests. 

## Figures and Tables

**Table 1:**
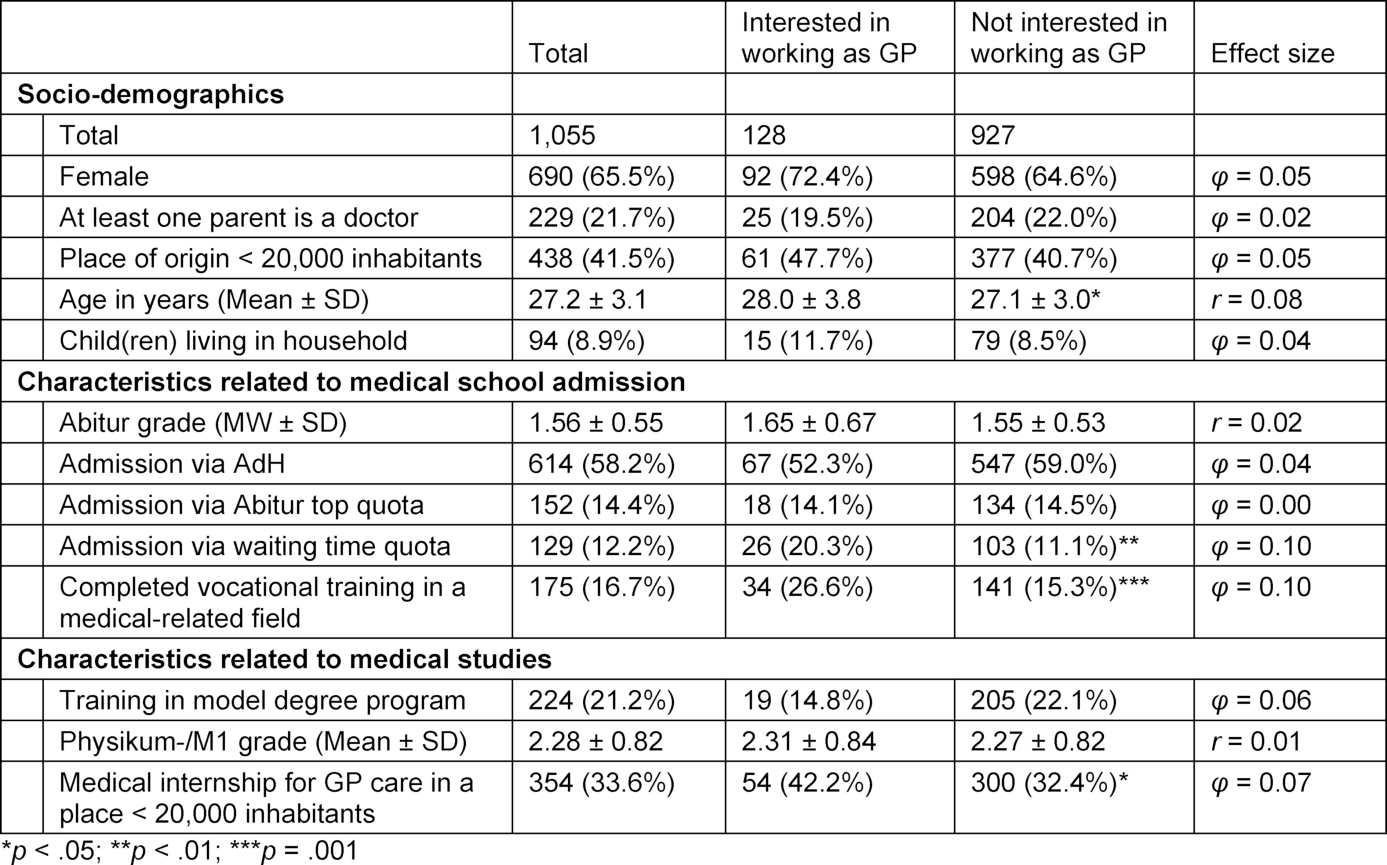
Study cohort according to interest in working as a GP

**Table 2:**
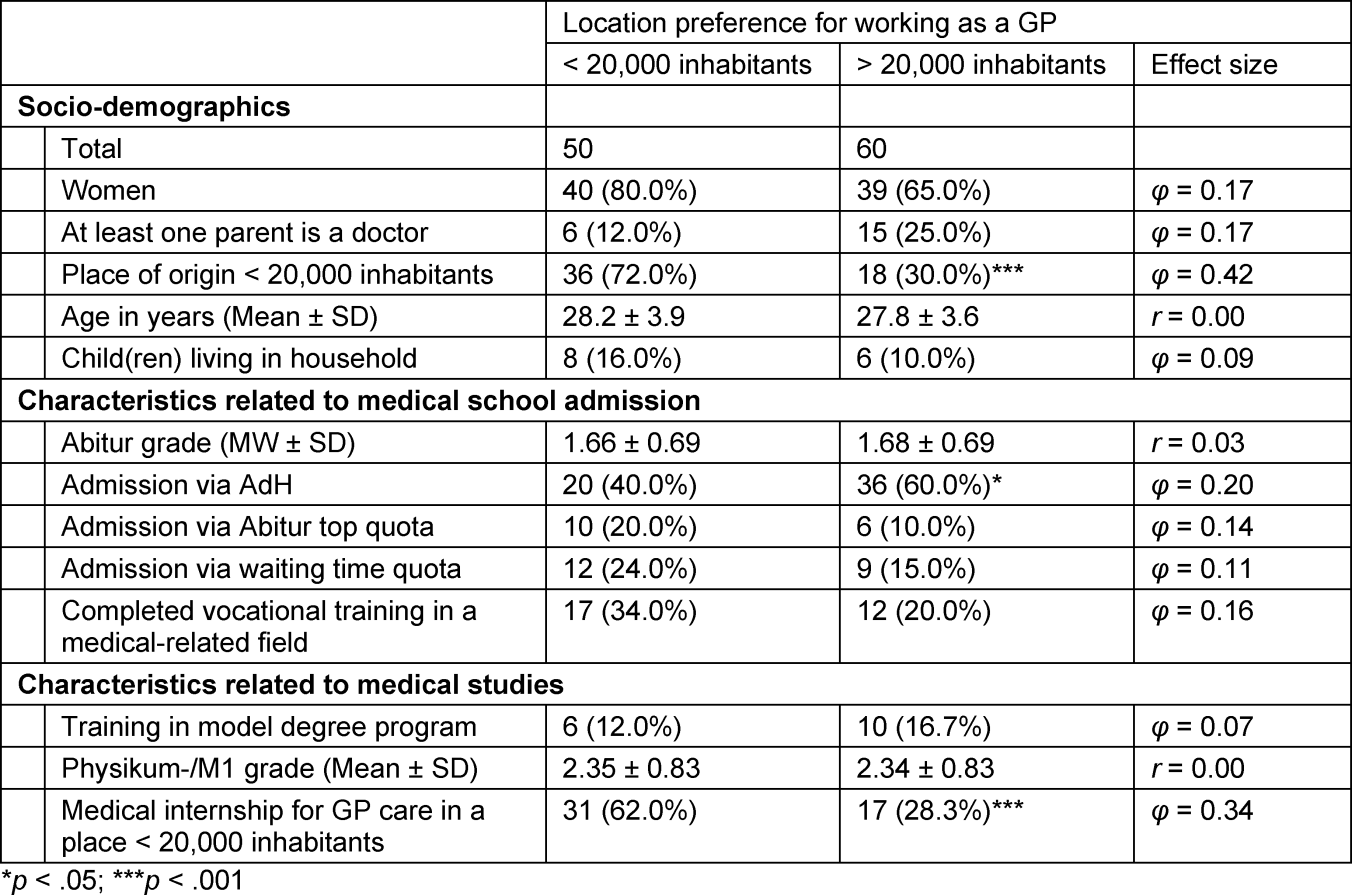
Students with an interest in working as a GP according to location preference

**Table 3:**
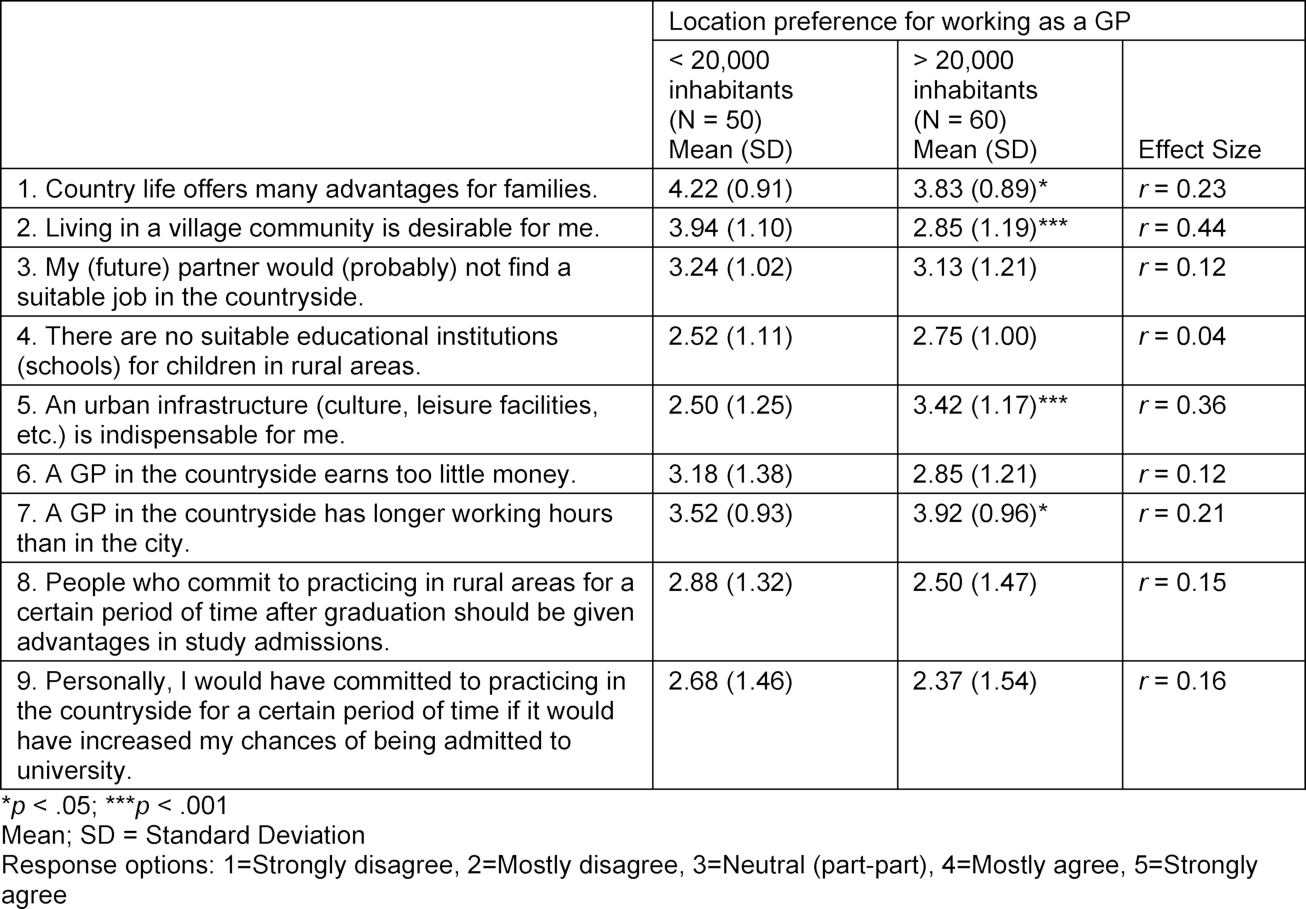
Attitudes towards life and working as a GP in rural areas
